# Assessment and Management of Frailty

**DOI:** 10.3390/jcm12030833

**Published:** 2023-01-20

**Authors:** Yuki Kato, Akio Shimizu, Ryo Momosaki

**Affiliations:** 1 Department of Rehabilitation, Mie University Hospital, Tsu 514-8507, Japan; 2 Department of Health Science, Faculty of Health and Human Development, The University of Nagano, Nagano 380-8525, Japan; 3 Department of Rehabilitation Medicine, Mie University Graduate School of Medicine, Tsu 514-8507, Japan

Frailty has become a major problem for an increasing number of older people worldwide. Frailty is defined as a reduced resilience to stress, due to an age-related loss of reserve capacity and is positioned as an intermediate state between healthy individuals and those in need of care [1]. Frail older adults are at a higher risk of hospitalization, death, and depression compared to healthy adults [2]. In addition, approximately 10% of older people in the community are frail [3], and frail older adults are five times more expensive to care for than non-frail older adults [4]. Frail older adults are expected to significantly increase in prevalence with the global aging of the population, causing a major public health issue [5].

Frailty overlaps with sarcopenia and malnutrition in many respects. Asian guidelines for frailty care identified aging, sarcopenia, polypharmacy, and malnutrition as contributing factors to frailty [6]. Additionally, social isolation and poverty-related diseases were associated with frailty [7]. However, many unknown factors that contribute to frailty remain, and further study is needed.

To assess frailty, several measurements and diagnostic tools can be used, but they are highly heterogeneous [8]. Validated assessment tools should be used when choosing an assessment tool for frailty. The two most widely used diagnostic and screening methods are the phenotype model (CHS criteria) [9] and cumulative deficit model [10]. The phenotype model of Fried et al. revealed weight loss, muscle weakness, exhaustion, slowness and low activity as the criteria for frailty. The model defines frailty in the following manner: the presence of one or two of these categories is defined as pre-frailty and the presence of three or more categories as frailty. The frailty component of the phenotype model has been incorporated into many questionnaire-based frailty screening tools. The FRAIL scale [11] and PRISMA-7 [12] are easy-to-use screening tools based on phenotype models. Screening tools that focus on social aspects, such as the place of residence, income, and financial management, have been developed, including the Tilburg Frailty Index [13] and the Vulnerable Elders Survey [14]. The Self-Rated Health Deficits Index [15] and the G8 Questionnaire [16] have been developed as questionnaires that include subjective health factors, such as how healthy individuals consider themselves compared to their past selves and others of the same age group.

The cumulative deficit model considers frailty as the accumulation of health problems. Approximately 30–70 health-related categories are defined, and the Frailty Index is calculated as the ratio of the number of applicable categories to the total number of categories. The Frailty Index is a continuous variable; thus, determining the severity of frailty is possible by setting a certain cut-off. The concept of the cumulative deficit model has been used in various countries and regions. For example, the electronic Frailty Index [17] that can be automatically calculated from comorbidities, daily living activities, and social problem categories in a specific database, the multimorbidity Frailty Index [18] that can be calculated from reimbursement databases, and other evaluation scores tailored to the situation and target patients have been developed. Some scores that can be automatically calculated from the collected information in electronic medical records were developed as more convenient frailty screening tools. The Hospital Frailty Risk Score is a score determined by weighting the International Classification of Diseases, Tenth Revision codes for comorbidities and can be automatically calculated in hospitals using electronic health records [19]. Various screening tools for frailty diagnosis have been devised, but a consensus has not been reached regarding the best setting in which to use these tools.

Management, including rehabilitation, is important for older patients with frailty, sarcopenia, and malnutrition. Rehabilitation and nutritional care have the potential to improve physical function, daily living activities, and quality of life in these older adults. Not many evidence-based treatments are available, although frailty is a reversible condition and is regarded as preventable and treatable. Strong recommendations in frailty management include the application of a progressive and individualized physical activity program, including resistance exercise, and the reduction in or discontinuation of inappropriate/unnecessary medications related to polypharmacy [6]. Additionally, providing energy and protein supplementation to frail older adults who show weight loss is effective if the cause is expected to be reversible after screening. Nutritional supplementation is more effective when combined with resistance exercise and other interventions. However, frail older adults and sarcopenia are often excluded from general clinical studies, and not many studies have been conducted for treatment purposes. Further study is needed on better methods of assessing malnutrition, sarcopenia, and frailty, and management strategies, including appropriate rehabilitation and nutritional support.
